# Traumatic Rupture of A Posterior Mediastinal Teratoma following Motor-Vehicle Accident

**DOI:** 10.1155/2016/7172062

**Published:** 2016-08-31

**Authors:** Christopher Bell, Fernando Domingo, Ashley D. Miller, Jeremiah S. Smith, James R. Headrick

**Affiliations:** Department of Surgery, University of Tennessee College of Medicine, 979 East Third Street, Suite C720, Chattanooga, TN 37404, USA

## Abstract

We report a case of a posterior mediastinal mature cystic teratoma with rupture secondary to blunt chest trauma in a 20-year-old male involved in a motor-vehicle accident. Initial treatment was guided by Advanced Trauma Life Support and a tube thoracostomy was performed for presumed hemothorax. The heterogeneous collection within the thoracic cavity was discovered to be the result of a ruptured cystic mass. Pathologic findings confirmed the mass consistent with a mature cystic teratoma. As mediastinal teratomas are most commonly described arising from the anterior mediastinum, the posterior location of the teratoma described in this report is exceedingly rare.

## 1. Introduction

Teratomas are considered the most common germ cell neoplasms and are characteristically found in the anterior-superior mediastinum [1]. The presence of teratomas in a posterior mediastinal location is rare. Teratomas are commonly asymptomatic and can be found incidentally on radiographic imaging [2]. Symptoms such as chest pain, dyspnea, cough, and pneumonitis may occur due to size of the tumor and the compressive mechanical effect of the neoplasm pushing against or invading into adjacent structures within the mediastinum [2]. Rapidly expanding tumors and inflammatory processes including infection, ischemia, and autolysis from tumor secretions can cause weakening of the cyst wall resulting in spontaneous rupture; however, ruptures secondary to trauma are rarely reported [3]. We report a rare case of a posterior mediastinal mature cystic teratoma with rupture due to blunt chest trauma from a motor-vehicle accident. The authors declare that there are no conflicts of interest regarding the publication of this case report.

## 2. Case Presentation

A 20-year-old, otherwise healthy, male was an unrestrained driver in a single-car rollover motor-vehicle accident. The patient was intubated on scene for airway protection and was then air-transferred to our facility. On initial assessment, the patient became hypoxic and hypotensive. A chest X-ray was obtained in the emergency room demonstrating a hemo/pneumothorax. This prompted a tube thoracostomy with immediate drainage of bloody, fat-laden, gelatinous, milky material. Hair was noted within the chest tube upon placement.

**Figure 1:**
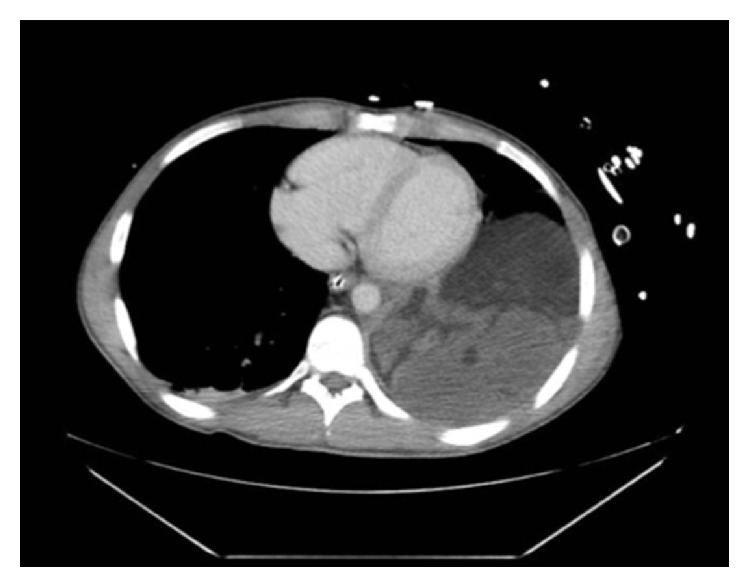
CT scan of the chest.

Once hemodynamically stable, the patient underwent computed tomography of his chest, abdomen, and pelvis which showed a large heterogeneous mixed density (fat, soft tissue, and calcifications) mass nearly completely replacing the left hemithorax (Figure 1). This extended from the superior mediastinum, inferior medially toward the left periaortic area. Additional injuries included a nondisplaced fracture of the left first rib, closed head injury, nondisplaced C7 fracture, and bilateral atelectasis.

**Figure 2:**
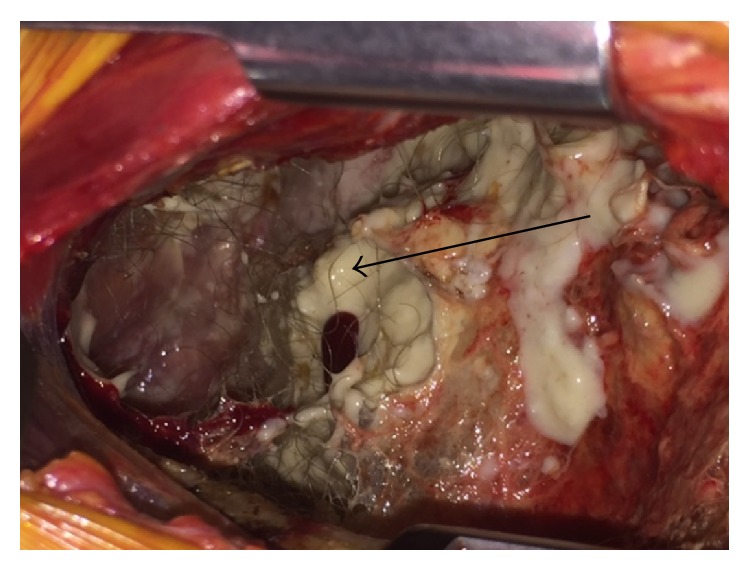
Image of thoracotomy incision demonstrating ruptured components of teratoma in pleural space. Arrow identifying hair.

**Figure 3:**
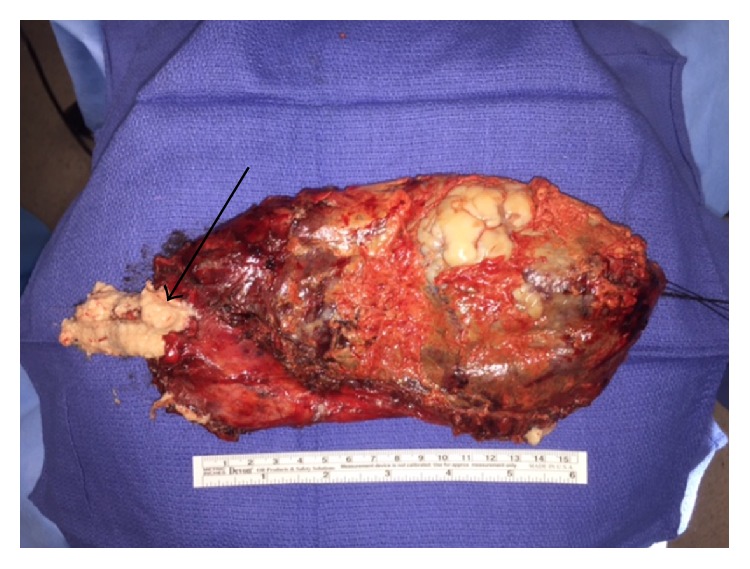
Image of teratoma. Arrow identifies the area of rupture.

After extubation and recovery from initial trauma, the patient was taken to the operating room on hospital day four for surgical intervention. Preoperative labs showed normal serum carcinoembryonic antigen (CEA) and alpha-fetoprotein (AFP) levels but an elevated serum CA 19-9. A standard left posterolateral thoracotomy was performed in the 6th intercostal space. Immediately upon entering the thoracic cavity, the patient was found to have gross contamination of hair and gelatinous white material within the pleural cavity that appeared to be the result of the ruptured cystic mass (Figure 2). This was evacuated from the thoracic cavity. The mass itself was found to be originating from the posterior mediastinum extending from the inferior pulmonary vein to below the diaphragm posteriorly. The mass displaced the left hemidiaphragm inferiorly and anteriorly. It extended down the aorta toward the esophagus, encasing several intercostal vessels. The superior aspect of the mass appeared to be missing a portion of the cyst wall with exposed gelatinous material and hair consistent with cyst rupture (Figure 3). The mass was resected en bloc. Due to its size and extension below the diaphragm, a second thoracotomy incision was made in the tenth intercostal space to facilitate removal. A new 32-French chest tube was placed prior to closure.

The tumor was grossly 20 × 10 × 3.5 cm in size, cystic in nature, and filled with abundant sebaceous material, hair, and fatty-appearing tissue. Pathology revealed the neoplasm to be a dermoid cyst, with a variety of associated tissues including skin adnexa, nerve/ganglion tissue, lymphoid nodules, bone, and a variety of glandular/parenchymal tissues, consistent with a diagnosis of mature cystic teratoma. Pathology did not show evidence of immaturity or malignancy. After an uneventful postoperative course, the patient was discharged home on the third postoperative day, following chest tube removal.

## 3. Discussion

Teratomas are germ cell tumors arising from pluripotent embryonic cells and typically originate in the gonads; however, the most common extragonadal site is the anterior-superior mediastinum [1, 3, 4]. As the majority of posterior mediastinal masses are typically neurogenic in origin, the posterior mediastinal teratoma described in this report is considered rare, as germ cell tumors of the mediastinum are classically found anteriorly [1]. Mature teratomas contain normal well-differentiated tissue elements derived from the ectodermal, mesodermal, and endodermal primitive embryonic layers and are found in an abnormal location. Mature teratomas are the most common primary mediastinal germ cell tumor [1, 3].

Most of these tumors are asymptomatic but are most commonly diagnosed in the 1st through 4th decade of life with mean age of presentation being 25–30 years [2, 3]. When symptoms are present, they are due to the mechanical mass effect on surrounding tissues causing chest pain, cough, dyspnea, and recurrent pneumonitis [1, 3]. Erosion into the tracheobronchial tree can lead to a productive cough with emission of hair and sebaceous material, referred to as trichoptysis [3, 4]. Surgical excision is necessary for appropriate diagnosis and is considered adequate therapy for most benign mature teratomas [1]. It is the treatment of choice in ruptured mediastinal teratomas [3]. Malignant and immature teratomas also require chemotherapy and radiotherapy in combination with surgical excision using an individualized approach for the varying types of malignant components [1–4].

Approximately 36% of all mediastinal teratomas are associated with rupture that is most frequently spontaneous in nature and can result in significant symptoms including severe chest pain, hemoptysis, fever, and severe respiratory distress [3, 5, 6]. It is hypothesized that spontaneous rupture is the result of digestive and proteolytic enzymes released from pancreatic tissue, salivary gland tissue, or intestinal epithelium [3–6]. Pancreatic tissue acts as a source of proteolytic enzymes and is commonly found in benign mature teratomas, which makes them more frequently associated with rupture than other teratomas [2, 3, 6]. This may correlate with the elevated serum CA 19-9 seen preoperatively in our patient. Other tumor markers, including CEA, AFP, lactate dehydrogenase, and beta-human chorionic gonadotropin, may be elevated but are more commonly associated with malignant and immature tumors [1].

Other causes of spontaneous rupture can be attributed to sebaceous secretions within the cyst, which can weaken the cyst wall and lead to rupture [1, 5, 6]. Infection can lead to rupture by increasing the fragility of the cyst wall [1, 5, 6]. Necrosis secondary to ischemia from rapidly expanding tumors has also resulted in spontaneous rupture [1, 5, 6]. Although some or all of these mechanisms may have caused an increased susceptibility for tumor rupture in our patient, very few reports have named trauma as the definitive cause of teratoma rupture as illustrated in this case [1, 7, 8]. Additionally, those reports describe the rupture of teratomas located in the more characteristic anterior mediastinum, while we report a rare case of the traumatic rupture of a mature teratoma originating from the posterior mediastinum.
